# A Biomimetic Electrochemical Approach for Chlorogenic Acid Detection in Herbal Supplements and Plant Extracts Using Molecular Imprinting Technology

**DOI:** 10.1021/acsomega.6c01396

**Published:** 2026-05-07

**Authors:** Seyda Yayla, Ahmet Cetinkaya, Engin Kocak, Emirhan Nemutlu, M. Mesud Hurkul, Sibel A. Ozkan

**Affiliations:** † Ankara University, Faculty of Pharmacy, Department of Pharmaceutical Botany, 06560 Ankara, Türkiye; ‡ University of Health Sciences, Gülhane Faculty of Pharmacy, Department of Analytical Chemistry, 06018 Ankara, Türkiye; § 37515 Hacettepe University, Faculty of Pharmacy, Department of Analytical Chemistry, 06100 Ankara, Türkiye; ∥ 37504 Ankara University, Faculty of Pharmacy, Department of Analytical Chemistry, 06560 Ankara, Türkiye

## Abstract

In this work, a novel molecularly imprinted polymer (MIP)-based electrochemical sensor was designed for the selective detection of chlorogenic acid (CGA), with the MIPs synthesized through a photopolymerization strategy employing 2-thienylboronic acid (2-TBA) as the functional monomer, thereby creating highly specific recognition sites capable of selectively binding CGA molecules within complex sample matrices. The applicability of the proposed MIP-based sensor was demonstrated by electrochemical detection of CGA in authentic samples of several Asyneuma species, including *Asyneuma limonifolium* and *Asyneuma linifolium* subspecies, indicating its potential for use in plant extract and herbal supplement analysis. A comprehensive evaluation of the CGA/2-TBA@MIP-GCE sensor was conducted to elucidate its morphological features and electrochemical characteristics, providing detailed insights into surface topography, electron-transfer kinetics, and overall sensor responsiveness. The analysis revealed that the imprinted polymer matrix exhibited highly organized recognition sites and efficient charge-transport pathways. For indirect CGA determination using 5.0 mM [Fe­(CN)_6_]^3–^/^4–^ as the redox probe, linearity was observed between 2.5 × 10^–13^ and 1.75 × 10^–12^ M. Based on the standard calibration data, the sensor exhibited an extremely high sensitivity, with a limit of detection (LOD) calculated at 2.34 × 10^–14^ M and a limit of quantification (LOQ) of 7.79 × 10^–14^ M. These results highlight the sensor’s remarkable ability to detect even trace amounts of CGA with precision and reliability. The developed electrochemical sensor based on molecularly imprinted polymers showed remarkable selectivity and sensitivity toward CGA, highlighting its robust molecular recognition properties. Furthermore, comprehensive recovery analyses were conducted to evaluate the platform’s practical applicability in real sample matrices, and the obtained recovery values were within acceptable analytical ranges. These results collectively indicate that the proposed sensor system functions as a robust, reliable, and analytically sensitive platform for the quantitative determination of CGA.

## Graphical abstract

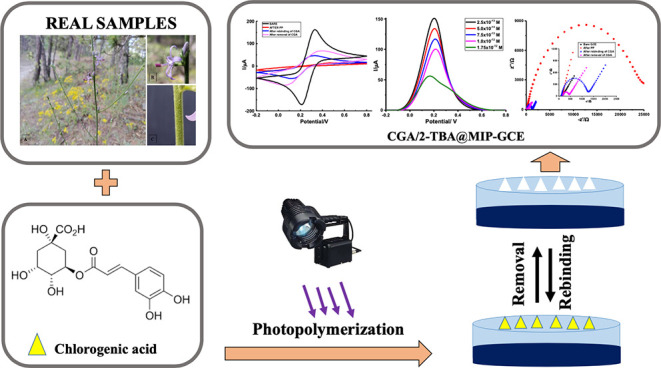

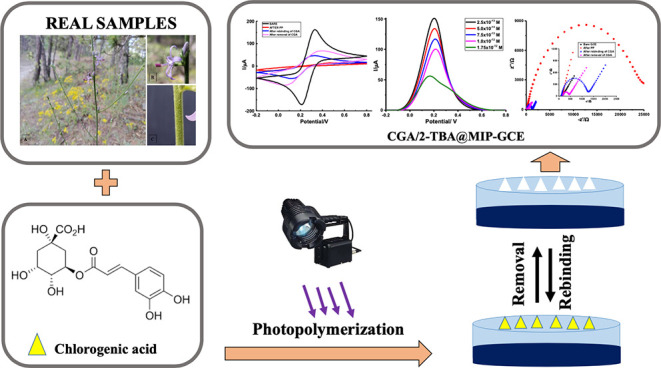

## 1 Introduction

Medicinal plants are valuable natural resources that have been used for therapeutic purposes since ancient times.^1,2^ The various parts of these plants, including leaves, flowers, roots, and seeds, play a crucial role in promoting health because they contain bioactive phytochemicals. According to data from the World Health Organization (WHO), 80% of people in developing countries use medicinal plants to meet their basic health needs.^3–5^ The Campanulaceae family includes many plants widely used in traditional medicine worldwide. *Campanula glomerata* Hegetschw. is used to treat diabetes, and *Campanula rapunculus* L. is used to treat stomach ailments in Türkiye.^6,7^ *Adenophora stricta* Miq. is consumed in China to aid weight loss.^8^ The Campanulaceae family is notable for its rich chemical composition. They contain a variety of bioactive substances, including phenolic compounds, flavonoids, triterpenoids, alkaloids, and saponins.^9^ In addition to their traditional uses, plants belonging to the Campanulaceae family show great promise in modern medical research. The bioactive compounds found in these plants may hold the key to developing new drugs.^10^

Chlorogenic acid (CGA) is a natural phenolic compound that is found in abundance in many plants, including green coffee beans, tea, and apples. Renowned for its antioxidant, anti-inflammatory, antimicrobial, antidiabetic, and antihypertensive properties, CGA plays a vital role in preventing and treating metabolic diseases. Studies in the literature have demonstrated that CGA is increasingly recognized as a bioactive polyphenolic ester with diverse pharmacological relevance, exerting regulatory control over central metabolic processes through modulation of glucose uptake, insulin signaling pathways, and lipid biosynthesis, which collectively influence the progression of obesity, type 2 diabetes mellitus, and nonalcoholic fatty liver disease. At the molecular level, CGA’s pronounced antioxidative capacity attenuates intracellular reactive oxygen species (ROS) accumulation. It ameliorates oxidative damage to macromolecules, a mechanism implicated in mitigating neurodegenerative cascades characteristic of Alzheimer’s and Parkinson’s diseases. Moreover, CGA’s ability to modulate redox-sensitive transcription factors and apoptotic regulators contributes to its observed chemopreventive effects across multiple cancer models. These mechanistic insights position CGA as a multifunctional phytochemical of significant therapeutic interest, with potential utility in targeted interventions against chronic metabolic dysregulation and degenerative pathologies. However, CGA also has limitations, such as low bioavailability and potential side effects (e.g., headaches and stomach upset) at high doses. Therefore, ongoing studies are investigating structural modifications and drug delivery systems to expand the clinical use of CGA. In addition to its use as a natural ingredient in the food industry, CGA also shows promise in pharmacological and nutraceutical applications.^11–15^

Molecularly imprinted polymer (MIP)-based electrochemical sensors are essential for analyzing plant extracts due to their high levels of selectivity, sensitivity, and speed. Plant extracts contain many similar chemical compounds, including phenolic acids such as CGA, and conventional analytical methods can make it challenging to accurately identify template molecules within these complex matrices. Thanks to their specific cavities, MIP-based sensors can distinguish template molecules from other compounds, providing specific analysis.^16–19^ Furthermore, these sensors are inexpensive, portable, and environmentally friendly, making them ideal for routine analysis of plant extracts.^20^ The quantification of biologically active compounds, such as CGA, is significant for the quality control and standardization of herbal products. This study demonstrates that MIP sensors can accurately determine CGA levels in plant extracts, thereby contributing to the development of safe and effective herbal supplements.

Recent progress in electrochemical sensor technology has markedly enhanced the sensitivity, selectivity, and response time of analytical platforms. Notably, sensors utilizing nanostructured materials and MIPs have garnered significant interest due to their high specific surface area, improved electron-transfer kinetics, and molecular recognition capabilities for target analytes. These advancements have facilitated the development of highly selective and sensitive electrochemical sensing systems capable of quantifying diverse analytes within complex matrices, underscoring their pivotal role in contemporary analytical methodologies. Furthermore, such innovations have substantially advanced analytical performance metrics, enabling rapid and precise detection. Specifically, the emergence of MIP-based electrochemical sensors, coupled with dual-signal electrochemical detection modalities, demonstrates considerable promise in enhancing analytical accuracy, robustness, and signal reliability in challenging sample environments.^21,22^

The genus *Asyneuma Griseb. & Schenk*, a member of the Campanulaceae family, is locally known as “*Çiçeklidegnek*” in Türkiye. It encompasses 34 species distributed across various regions of the world, exhibiting considerable morphological diversity in terms of vegetative and reproductive traits. Species within this genus are typically perennial herbs with characteristic inflorescence and corolla structures, which are taxonomically significant. The Campanulaceae family, to which Asyneuma belongs, has been extensively documented for its ethnobotanical applications; plants of this family have traditionally been used in different cultures to manage a wide range of ailments, reflecting their importance in both folk medicine and contemporary phytochemical studies.^10^ This study describes the development of a molecularly imprinted polymer (MIP)-based electrochemical sensor designed for the selective and highly sensitive determination of CGA in extracts obtained from various Asyneuma subspecies, including *Asyneuma limonifolium* subsp. *limonifolium*, *Asyneuma limonifolium* subsp. *pestalozzae* (Boiss.) Damboldt, *Asyneuma linifolium* subsp. *linifolium*, and *Asyneuma linifolium* subsp. *nallihanicum*. The sensor’s analytical performance was further validated by its application to commercially available herbal supplement products, demonstrating its suitability for precise CGA quantification in both natural and commercial matrices. The sensor was constructed via photopolymerization (PP), utilizing 2-thienylboronic acid (2-TBA) as the functional monomer, 2-hydroxyethyl methacrylate (HEMA) as the basic monomer, and ethylene glycol dimethacrylate (EGDMA) as the cross-linking agent. Comprehensive morphological characterization was performed using scanning electron microscopy (SEM) coupled with energy-dispersive X-ray spectroscopy (EDX) to confirm the sensor’s structural integrity and surface features. Electrochemical performance was systematically evaluated through cyclic voltammetry (CV) and electrochemical impedance spectroscopy (EIS). Furthermore, the sensor’s applicability to real sample matrices was validated through recovery studies, highlighting its potential for precise quantification of CGA in complex herbal extracts and supplements.

## 2 Experimental Section

### 2.1 Reagents and Chemicals

The chemicals including CGA (≥95.0%), *p*-coumaric acid (p-CA, ≥98.0%), caffeic acid (CAF, ≥98.0%), apigenin (API, ≥95.0%), luteolin (LUT, ≥98.0%), hyperoside (HYP, ≥95.0%), quercetin (QUE, ≥95.0%), rutin hydrate (RUT, ≥94.0%), naringenin (NAR, 98.0%), gallic acid (GAL, ≥95.0%), methanol (MeOH, ≥99.8%), acetone (≥99%), sodium hydroxide (NaOH, ≥97.0%), acetic acid (HAc, ≥99.0%), ethanol (EtOH, ≥99.0%), ethylene glycol dimethacrylate (EGDMA, >98.0%), 2-hydroxyethyl methacrylate (HEMA, ≥99%), acetonitrile (ACN, ≥99.9%), 2-hydroxy-2-methyl propiophenone (≥97%) used in the experiments were purchased from Sigma-Aldrich (St. Louis, Missouri, USA), and potassium salts of ferricyanide (K_4_[Fe­(CN)_6_]·3H_2_O; ≥99.0%) and ferrocyanide (K_3_[Fe­(CN)_6_]; ≥98.5%) were purchased from Sigma-Aldrich (St. Louis, Missouri, USA). The herbal supplement containing the active compound CGA was purchased from a local pharmacy. The analyzed plants (*A. limonifolium* subsp. *limonifolium*, *A. limonifolium* subsp. *pestalozzae*, *A. linifolium* subsp. *linifolium*, *A. linifolium* subsp. *nallihanicum*) were collected from their natural habitats.

### 2.2 Equipment/Apparatus

Weighing was conducted using a precision balance (Ohaus Instruments, model PX2201, Shanghai, China), and pH adjustments were made with a pH meter (Mettler-Toledo pH/ion S220, Switzerland). For photopolymerization, a 100 W UV lamp (365 nm) was used. The electrochemical measurements were performed using a three-electrode system: (1) a glassy carbon electrode (GCE, outer diameter 3.0 mm) as the working electrode; (2) an Ag/AgCl (3 M KCl) reference electrode; and (3) a platinum wire as the counter electrode. A PalmSens Potentiostat (PSTrace 5, Netherlands) was used for cyclic voltammetry (CV) and differential pulse voltammetry (DPV) measurements. AUTOLAB (Nova 2.1.5 software, Netherlands) was used for electrochemical impedance spectroscopy (EIS) analysis. During sensor development studies, removal and rebinding operations were performed using a Thermo-shaker (Biosan TS-100) to maintain constant temperature. An ultrasonic bath (JP Selecta Corporation, Barcelona, Spain) and a vortex (ISOLAB Laborgeräte GmbH, Germany) were used to mix the solutions and prepare plant extracts homogeneously. Additionally, a rotary evaporator (BUCHI, Switzerland) was used to prepare plant extracts. The scanning electron microscopy (SEM) measurements for surface characterization were performed using a TESCAN GAIA 3 scanning electron microscope (Brno-Kohoutovice, Czech Republic).

### 2.3 Preparation of Electrode Surface

The glassy carbon electrode (GCE) was initially ultrasonically cleaned in a 1:1 (v/v) mixture of methanol and water for 15 min to remove surface contaminants. After sonication, the electrode was polished using a polishing pad embedded with 1.0 μm alumina particles, rinsed thoroughly with deionized water, and then dried at room temperature. The prepolymerization solution was prepared by combining 20 μL of the template molecule (CGA, 1 mM) with 40 μL of the functional monomer (2-TBA, 1.0 mM). To this mixture, 100 μL of 2-hydroxyethyl methacrylate (HEMA) as the basic monomer and 20 μL of ethylene glycol dimethacrylate (EGDMA) as the cross-linking agent were added. The resulting solution was sonicated for 15 min to ensure thorough mixing, and an initiator solution containing 2 μL of 2-hydroxy-2-methylpropiophenone was added to the prepolymerization mixture. Next, 0.25 μL of the vortexed mixture was deposited precisely onto the surface of the glass carbon electrode (GCE). The photopolymerization process was then carried out by exposing the coated electrode to ultraviolet irradiation at 365 nm with a power intensity of 100 W for 10 min at ambient temperature. The electrodes were removed from the UV light source and allowed to cool to room temperature for 15 min. The modified GCE sensor was then operated in a 15 M HAc solution at 25 °C and 650 rpm using a Thermo-shaker for 10 min to reveal specific cavities in the MIP structure. Finally, rebinding was performed for 20 min at 250 rpm and 25 °C using a Thermo-shaker, with CGA solutions of known concentration used to rebind the CGA molecules into the specific cavities. For control investigations, nonimprinted polymers (NIPs) were synthesized under the same experimental conditions and following the same protocol as for MIPs, with the sole deviation being the exclusion of the template molecule, CGA, from the prepolymerization mixture. This strategy ensures the formation of a polymer matrix devoid of CGA-specific recognition cavities, thereby serving as an appropriate control for assessing the selectivity, binding affinity, and overall analytical performance of the CGA-imprinted electrochemical sensors. The preparation process of the CGA/2-TBA@MIP-GCE sensor is illustrated in Scheme 1.

**Scheme 1:**
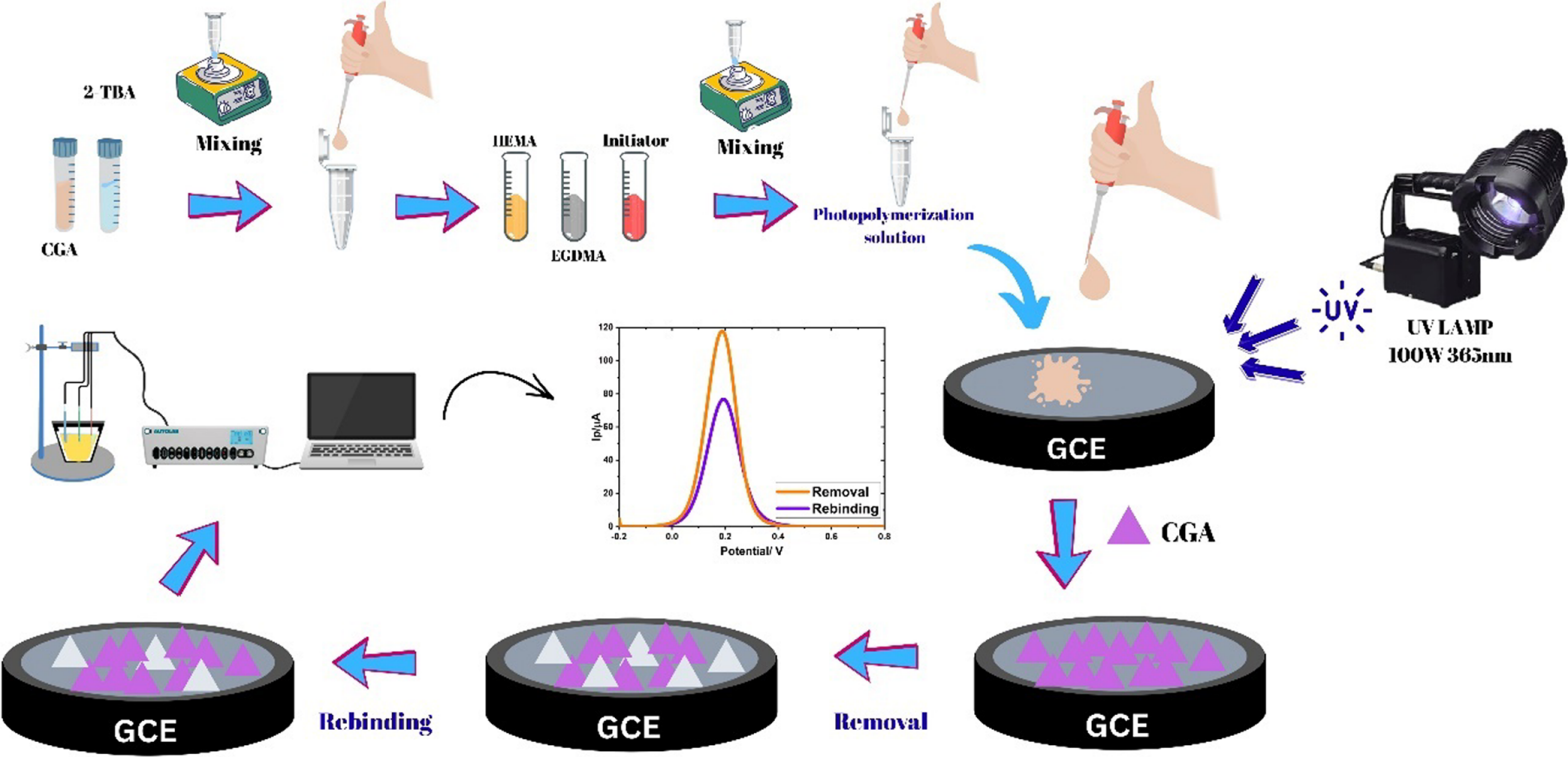
Preparation Process of the CGA/2-TBA@MIP-GCE Sensor

### 2.4 Preparation of Real Samples

Before beginning the experimental studies, the dried plant material was pulverized. MeOH extracts were prepared from the powdered samples. These were then heated in a 240-W ultrasonic bath at room temperature for 15 min. The extraction temperature was controlled by adding ice to the ultrasonic bath as needed and monitored with a bath thermometer. The prepared plant extracts were then used as the main stock solution. As the concentration of CGA in the extracts was unknown, intermediate stock solutions were prepared by diluting the extracts 1:100 with MeOH. Both the primary and intermediate stock solutions were stored in the refrigerator at 4 °C.

Five commercially available tablets, each containing 30 μg of CGA, were first ground to a fine powder using a porcelain mortar and pestle. A precise portion of this powder, calculated to yield a 1.0 mM CGA stock solution, was weighed on a high-accuracy analytical balance. The weighed powder was dissolved in methanol (MeOH) and sonicated for 30 min to achieve complete dissolution and a uniform solution. Following this, the mixture was centrifuged at 5000 rpm for 25 min to remove undissolved solids and particulate matter. The clear supernatant was collected as the primary stock solution, and intermediate working solutions were subsequently prepared by appropriate dilution with MeOH to obtain the desired concentrations for further analysis. These intermediate stock solutions were used to calculate the amount of CGA in tablet form during recovery studies using standard calibration data. Recovery studies were conducted by adding a specific amount of the pure CGA active ingredient to tablet samples of known concentration to examine the accuracy and reproducibility of the CGA content in the tablets. The CGA recovery rate was then calculated.

### 2.5 LC-MS/MS Parameters

The Shimadzu LC–MS/MS system (LC-20AXR coupled with a triple quadrupole mass spectrometer, LCMS-8030; Shimadzu, Japan) was employed for quantitative analysis, with the method adapted and optimized based on Cybulska et al. Chromatographic separation was achieved on a Discovery HS C18 analytical column (75 × 4.6 mm, 3 μm particle size) maintained at 40 °C. The mobile phase consisted of water containing 0.1% formic acid (solvent A) and acetonitrile containing 0.1% formic acid (solvent B). The flow rate was set to 0.4 mL min^–1^ with a linear gradient program as follows: 5–60% B from 0.0–2.0 min, held at 60% B for 1 min (2.0–3.0 min), decreased to 5% B within 1 min (3.0–4.0 min), and equilibrated at the initial conditions for 1 min (4.0–5.0 min). The total run time was 5 min, and the injection volume was 10 μL.

Detection was performed using electrospray ionization in positive ion mode with multiple reaction monitoring (MRM) for quantitative confirmation. The optimized MS parameters were as follows: interface voltage, 4.5 kV; collision energy, −14 V; desolvation line temperature, 250 °C; heat block temperature, 400 °C; nebulizing gas flow, 3 L min^–1^; and drying gas flow, 15 L min^–1^. Compound-specific parameters, including precursor and product ions, Q1/Q3 voltages, and collision energies, were optimized via direct infusion of standard solutions. For chlorogenic acid, the MRM transition of *m*/*z* 353 → 191 was monitored with a collision energy of 27 eV and Q1/Q3 voltages of 27 V. The dwell time for each transition was 100 ms.

Quantification of CGA was performed using an external calibration curve prepared from a 1000 ppm stock standard solution in MeOH. Working solutions were diluted in series to obtain five calibration levels ranging from 0.0625 to 1.0 μg mL^–1^.

## 3 Results and Discussion

### 3.1 Morphological Characterization of the Sensor Surface

The surface properties of the fabricated CGA/2-TBA@MIP-GCE sensors were thoroughly examined to assess their morphological characteristics. High-resolution scanning electron microscopy (SEM) was used to investigate the electrode surfaces in detail. SEM images revealed notable differences between the molecularly imprinted polymer (MIP) and nonimprinted polymer (NIP) electrodes, with the MIP surfaces exhibiting a more porous and well-organized structure, indicative of the successful formation of specific recognition sites for CGA molecules. These structural variations are crucial, as they directly affect the accessibility and distribution of the binding cavities, which underpin the sensor’s selectivity. In contrast, the NIP surfaces displayed a relatively smooth and featureless morphology, serving as an appropriate control for comparison. Based on the fabrication process, the MIP-modified electrode surfaces exhibit a rough and highly porous morphology, as illustrated in Figure 1A,1B. In contrast, the SEM micrographs of the NIP-based electrodes (Figure 1C,1D) show a predominantly smooth surface with no discernible porosity. This comparison highlights the formation of well-defined, selective binding cavities in the MIP surfaces, which are absent in the NIP controls, thereby confirming the imprinting effect and the structural basis for the sensor’s selectivity toward chlorogenic acid. These results clearly demonstrate that polymerization primarily depends on the presence of template molecules, which orient themselves toward the polymeric network and alter polymer film formation on the electrode surface. Moreover, these differences demonstrate the influence of template molecules on the formation of the polymeric network and its surface properties. EDX analysis was also used to characterize the surface of the CGA/2-TBA@MIP-GCE sensor. Figure 1E,1F demonstrate that the polymeric matrix of the modified sensor comprises carbon (C), oxygen (O), and gold (Au) atoms, as revealed by the EDX spectra. While the spectra of both the MIP and NIP surfaces share common chemical elements, their topographies differ. This is because polymerization occurs around template molecules in the MIP version. Despite the similar chemical composition of the polymeric network, these results reveal morphological differences on the MIP surface, which is a key indicator of the formation of selectively imprinted cavities. This clearly demonstrates the contribution of these structures to sensor performance.

**Figure 1:**
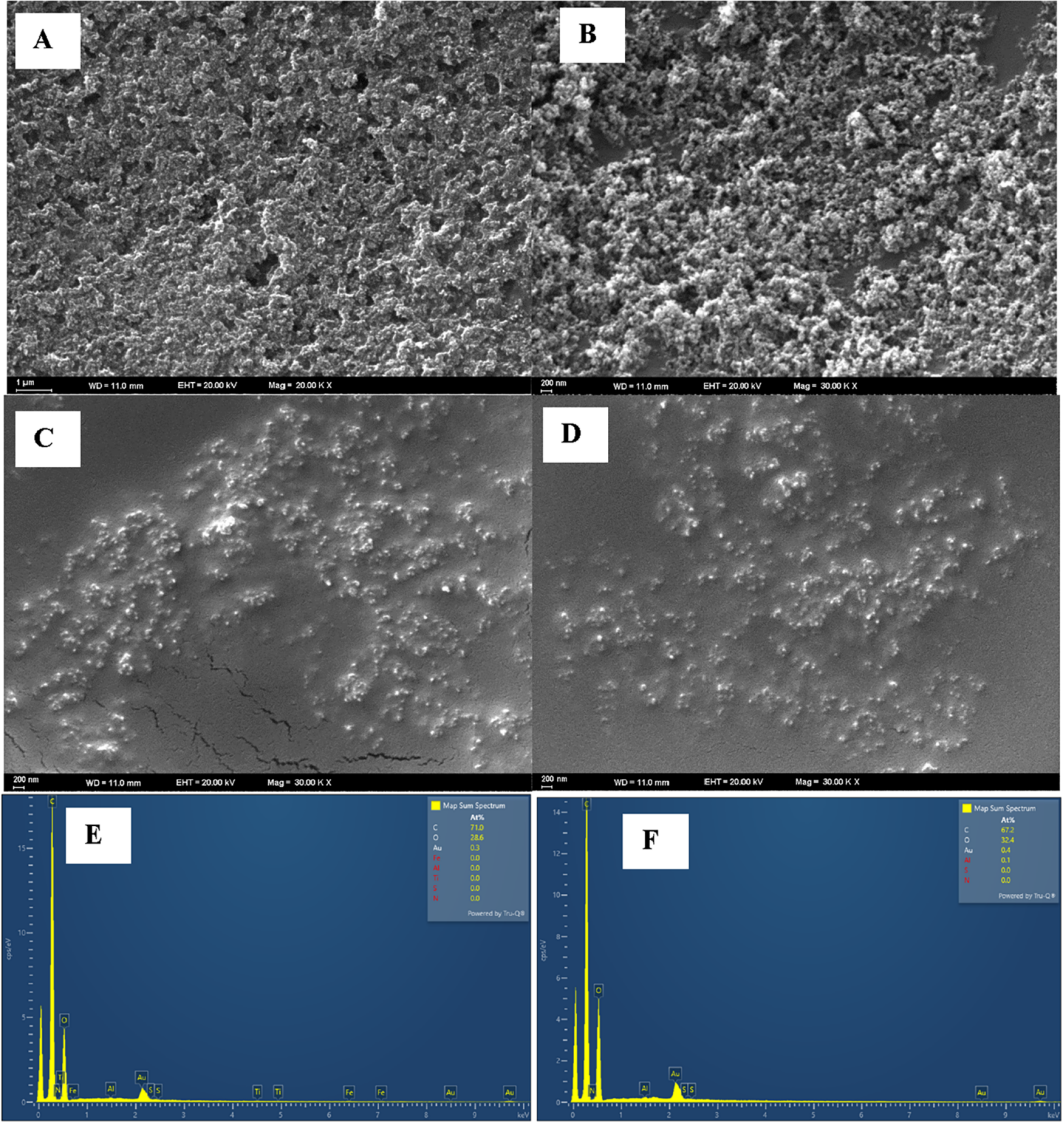
Surface characterizations of the CGA/2-TBA@MIP-GCE sensor. SEM images of (A, B) MIP and (C, D) NIP surfaces of CGA/2-TBA@NIP-GCE; and EDX spectra of (E) MIP and (F) NIP.

### 3.2 Electrochemical Characterization of CGA/2-TBA@MIP-GCE Surface

The electrochemical properties of the CGA/2-TBA@MIP-GCE sensor were thoroughly investigated using CV and EIS, which serve as essential methods for probing the electrode’s conductivity, charge-transfer resistance (Rct), and electron transfer kinetics at the interface. To facilitate these analyses, a 5.0 mM [Fe­(CN)_6_]^3–^/^4–^ redox probe was employed, allowing for systematic monitoring of the sensor’s electrochemical response throughout the fabrication process. Distinct changes in peak currents and interfacial resistance were observed at successive stages, including the bare GCE, after PP, template removal, and target analyte rebinding, reflecting the progressive modification of the electrode surface and the formation of specific recognition sites. The corresponding CV and EIS spectra, shown in Figure 2, illustrate the evolution of the sensor’s electrochemical behavior and confirm that the imprinting process effectively enhances selective electron-transfer pathways, which are critical for high-performance detection of CGA. In the CV plot (Figure 2A), maximum peak current values were obtained for the [Fe­(CN)_6_]^3–^/^4–^ redox probe (***black line***) because no factor inhibited electron transfer on the bare GCE surface. After polymerization, the GCE surface was coated with a polymeric film that blocked electron transfer, leading to a significant decrease in peak current. The peak current value of the [Fe­(CN)_6_]^3–^/^4–^ redox probe could not be observed (***red line***). After the CGA molecule was removed from the polymeric film, specific CGA-specific vacancies were formed on the surface, and the peak current value of the [Fe­(CN)_6_]^3–/4–^ redox probe was lower than that of the bare GCE (***purple line***). In this case, the peak current values were lower than those for the bare GCE but higher than those in the post-PP stage. In the final stage, when certain concentrations of CGA rebinding occur, the peak current decreases again due to partial closure of vacancies in the polymeric film. The resulting peak current value was lower than that obtained after removal of the template molecule (***blue line***).

**Figure 2:**
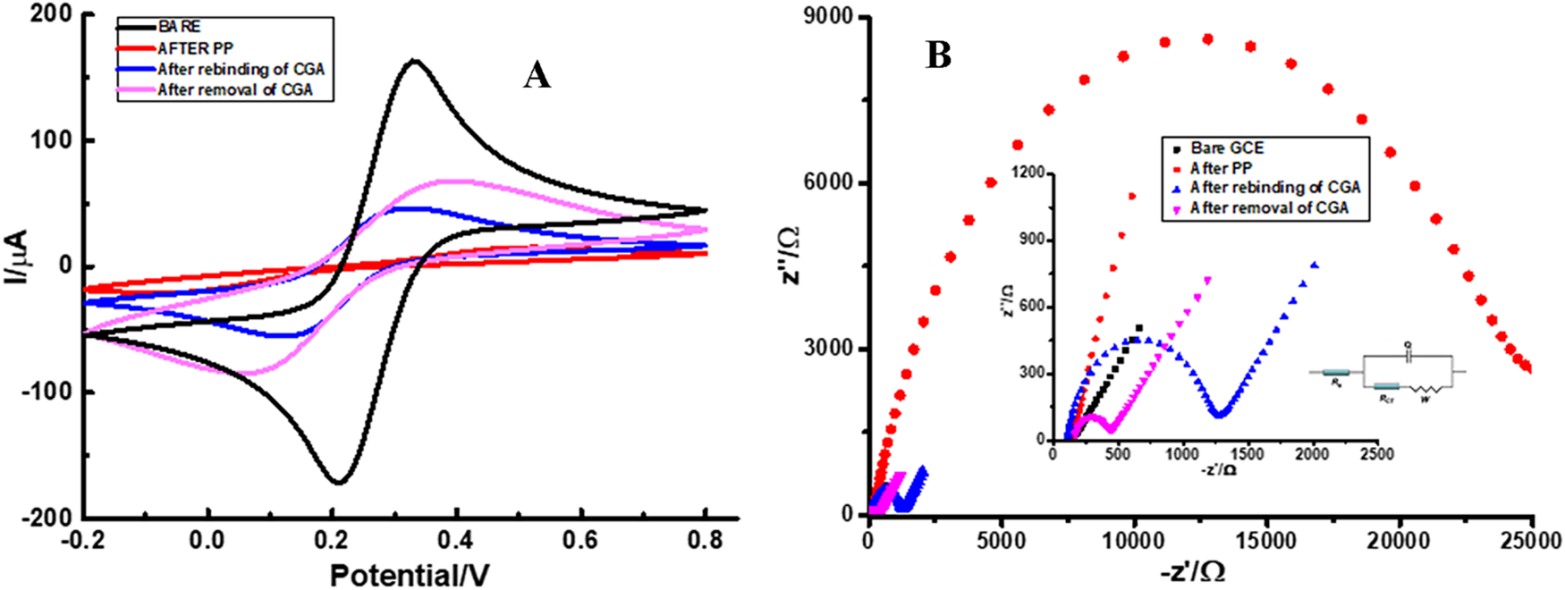
Electrochemical characterization of the designed CGA/2-TBA@MIP-GCE sensor: (a) cyclic voltammogram and (b) EIS, Nyquist plot.

Changes in Nyquist plots and *R*ct were examined in EIS measurements (Figure 2B and Table 1). The EIS data indicate that the bare GCE exhibited minimal charge-transfer resistance (*R*_ct_), as indicated by the ***black dots***, reflecting rapid, unhindered electron transfer at the unmodified electrode surface. Following polymerization, the polymeric layer deposited on the electrode resulted in a substantial increase in *R*_ct_, as evidenced by the ***red dots***, indicating that the polymer film effectively impedes electron mobility. This pronounced difference between the ***black*** and ***red dots*** clearly illustrates the impact of the polymer coating on interfacial electron transfer and confirms the successful establishment of the imprinted polymeric architecture on the GCE surface. The removal of CGA molecules led to electron transfer through specific vacancies on the MIP surface, resulting in a slight decrease in *R*_ct_ values (***purple dots***). However, when CGA reattached, it partially filled these vacancies, making electron transfer more difficult. This led to a return to higher *R*ct values (***blue dots***). These results clearly demonstrate that CGA molecules bind selectively to specific surface vacancies on the developed MIP sensor.

**Table 1:** *R*_ct_ Values Acquired at Different Stages of the Fabrication Process for the CGA/2-TBA@MIP-GCE Sensor

MIP stages	*R*_ct_ value/Ω
bare electrode	231.00
after PP	8450.00
after the removal of CGA	437.00
after the rebinding of CGA	898.00

In addition, using the peak current values obtained from GCE before polymerization, after polymerization, after removal, and after rebinding, the electroactive surface areas of GCE were calculated via the Randles–Sevcik equation (*I*_p_ = 2.69 × 105 *n*3/2*AD*1/2υ1/2*C*).^23^ In this equation, *I*_p_ stands for the peak current, *n* stands for the number of transferred electrons (calculated as 1 for potassium ferri/ferrocyanide), *A* stands for the active surface area (cm^2^), *D* stands for the diffusion coefficient (calculated as 7.6 × 10^–6^ cm^2^ s^–1^ for [Fe­(CN)_6_]^3–/4–^), υ stands for the scan rate, and *C* stands for the concentration of probe. The electroactive surface areas of the GCE were found to be 0.1359 cm^2^ before polymerization, 0.0104 cm^2^ after polymerization, 0.0555 cm^2^ after removal, and 0.038 cm^2^ after rebinding. These results confirm the successful formation of the polymer layer, the creation of accessible cavities after template removal, and the partial blocking of the surface during the rebinding process.

### 3.3 Optimization Parameters

#### 3.3.1 Template/Monomer Ratio

The optimization of the monomer-to-template ratio is a pivotal factor in the fabrication of stable and high-performance polymeric films. This ratio dictates the strength and specificity of interactions between the monomer and the template, which directly govern the formation and structural fidelity of the polymeric network. In molecular imprinting applications, an appropriately balanced monomer-to-template ratio is essential for enhancing the selectivity of the resulting sensor, ensuring that the polymer matrix preferentially accommodates the target analyte. Conversely, an excessive proportion of monomer may generate nonspecific regions within the polymer, compromising both the selectivity and structural stability of the film. To systematically investigate the effect of this parameter, polymeric films were prepared via photopolymerization with a range of monomer-to-template ratios (1:1, 2:1, 3:1, 4:1, and 5:1), enabling assessment of optimal conditions for efficient imprinting and sensor performance. Evaluation was made based on the difference (Δ*I*_1_) between the peak current values obtained after the template molecule was removed from the surface and those obtained after PP (Figure 3A). Following analyses to obtain the most effective and stable polymers, the monomer: template molecule ratio was optimized based on the Δ*I*_1_ values obtained with PP. The best value, 2:1 ratio, was selected for the study. The potential interactions between well-matched functional groups of CGA and the functional monomer (2-TBA) were considered, and the structural features were chosen to create a complementary structure, similar to a lock-and-key relationship, to achieve the highest level of specificity and selectivity. The results demonstrated the positive impact of the proposed ratios on the polymers’ stability and activity.

**Figure 3:**
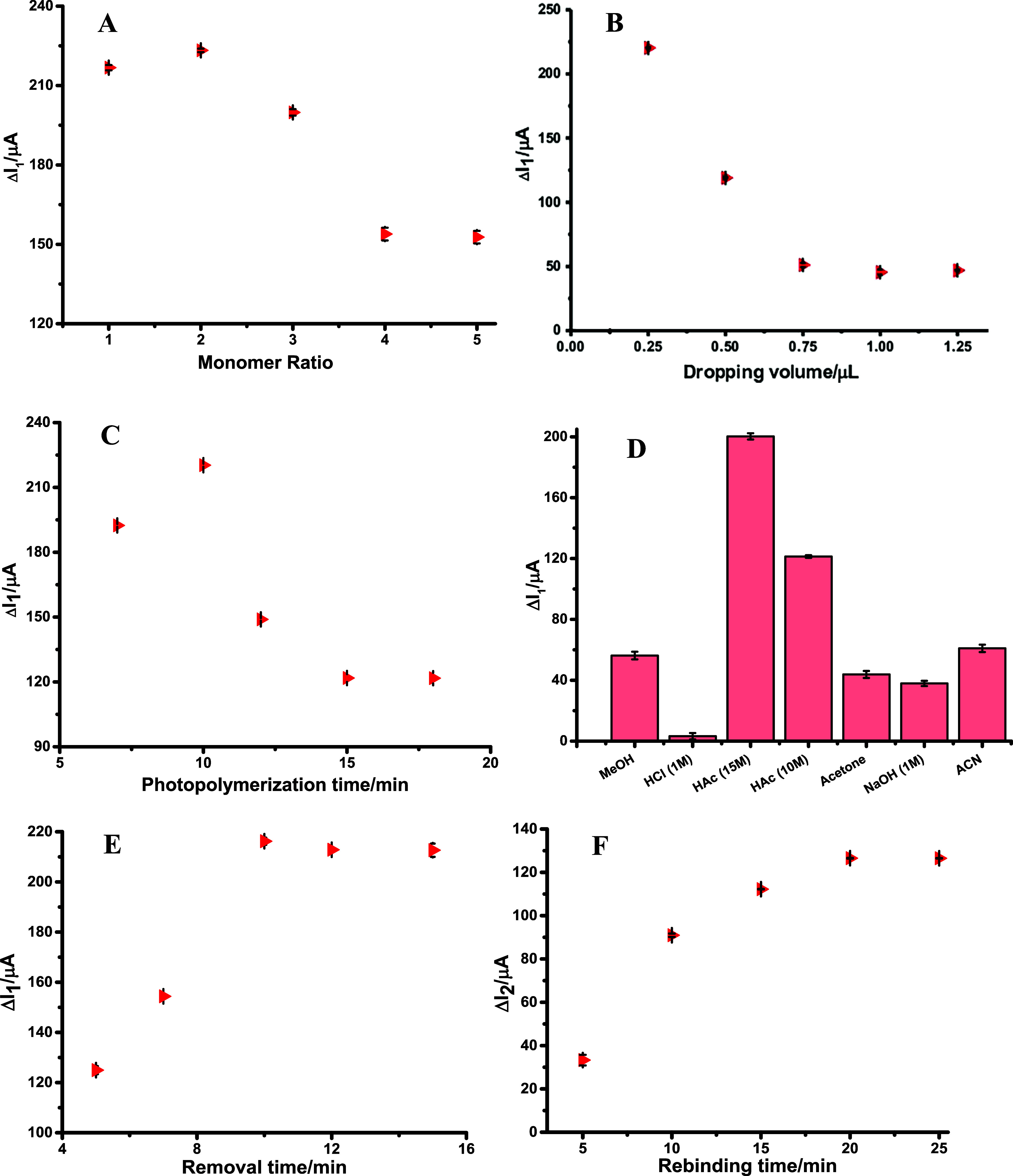
Optimization of (a) monomer/template ratio, (b) dropping volume, (c) PP time, (d) removal solution, (e) removal time, and (f) rebinding time of CGA/2-TBA@MIP-GCE in 5 mM [Fe­(CN)_6_]^3–/4–^ solution (0.1 M KCl).

#### 3.3.2 Dropping Volume

The volume of polymerization solution applied to the GCE surface represents a critical parameter that must be carefully optimized, as it directly influences the resulting polymeric film’s thickness, uniformity, and overall polymerization efficiency. Variations in the volume of the dropped solution can alter the morphology and electrochemical performance of the film, thereby affecting diffusion characteristics, conductivity, and sensor responsiveness. To determine the optimal conditions, different aliquots of polymerization solution (0.25, 0.5, 0.75, 1.0, and 1.25 μL) were systematically applied to the electrode surface. The CGA/2-TBA@MIP-GCE sensor was then evaluated under these conditions to identify the volume that yielded a polymeric film with the best compromise between structural homogeneity, minimal diffusion limitations, and high electrical conductivity. The differences in peak current (Δ*I*_1_) after the template molecule was removed from the GCE surface and after PP were evaluated, and the optimum dropping volume was found to be 0.25 μL (Figure 3B).

#### 3.3.3 PP Time

The fabrication of the CGA/2-TBA@MIP-GCE sensor was carried out under UV irradiation using a 365 nm lamp with a power output of 100 W. Careful optimization of the polymerization time is essential to achieve a uniform, mechanically stable polymeric film on the electrode surface. To this end, 0.25 μL of the polymerization solution was deposited onto the GCE, and the modified surface was subsequently exposed to UV light for 7, 10, 12, 15, and 18 min, respectively. This systematic variation allowed the determination of the optimal polymerization time that ensures both homogeneity and structural integrity of the imprinted polymer layer. The differences in peak current (Δ*I*_1_) were then compared after the target molecule and the PP were removed from the surface. Because the sensor preparation time should be kept as short as possible, the optimal polymerization time was set at 10 min (Figure 3C). Longer times can adversely affect the structure of the polymeric film, leading to diffusion limitations and conductivity losses.

#### 3.3.4 Removal Solutions and Time

Efficient extraction of target molecules from the MIP surface while preserving the structural integrity of the polymeric film is essential for generating well-defined recognition cavities that enable selective binding of analytes. In this part, various solutions were tested to remove template molecules from the surface. For the removal of CGA from the modified sensor, solutions of 10 and 15 M HAc, MeOH, 1.0 M NaOH, 1.0 M HCl, acetone, and ACN were used as removal agents. Based on the experimental findings, a 15 M HAc solution was identified as the most effective medium for extracting target molecules from the MIP surface (Figure 3D). To investigate the removal process in detail, the modified CGA/2-TBA@MIP-GCE sensor was immersed in HAc solution and incubated under controlled conditions using a Thermo-shaker at 650 rpm and 25 °C for 5, 7, 10, 12, and 15 min. The efficiency of template extraction was quantitatively assessed by monitoring changes in the peak current (Δ*I*_1_), defined as the difference between the sensor’s electrochemical response before and after the removal step. Analysis of the Δ*I*_1_ values indicated that a 10 min incubation period provided the most effective removal of the template molecules (Figure 3E). This result highlights that both the concentration of the removal solution and the exposure time are critical parameters for achieving complete extraction without compromising the integrity of the polymeric film, ensuring the formation of well-defined recognition cavities for subsequent analyte binding.

#### 3.3.5 Rebinding Time

Rebinding time is a key factor that significantly affects both the efficiency of analyte capture and the overall analytical throughput of MIP-based sensors. Inadequate rebinding periods may lead to incomplete occupancy of the imprinted cavities, reducing the sensor’s sensitivity, whereas excessively long exposure can prolong analysis without additional gains in binding efficiency. To systematically investigate this parameter, the CGA/2-TBA@MIP-GCE sensor prepared via photopolymerization was incubated in a 1.0 × 10^–10^ M chlorogenic acid solution for 5, 10, 15, 20, and 25 min. The incubation was performed under mild agitation using a Thermo-shaker set at 250 rpm and 25 °C, ensuring uniform interaction between the analyte molecules and the imprinted sites. Monitoring the sensor’s response across these time intervals allowed the determination of the period required for maximal rebinding, balancing efficient template recognition with practical analysis times. These observations underscore the importance of carefully optimizing rebinding duration to achieve both high selectivity and reproducible performance in molecularly imprinted electrochemical sensors. The results of the experiments were calculated as the difference between the peak currents (Δ*I*_2_) obtained after template molecule removal and rebinding. As shown in Figure 3F, the reconnection time for the CGA/2-TBA@MIP-GCE sensor was 20 min.

### 3.4 Analytical Performance Evaluation of MIP-GCE Sensor

In the analytical application of the fabricated electrochemical sensor (CGA/2-TBA@MIP-GCE), the main objective was to evaluate its performance for the quantitative determination of active pharmaceutical ingredient (CGA) from standard solutions. In this step, electrochemical measurements were performed using DPV, and a calibration curve was generated under optimized experimental conditions, enabling the evaluation of the sensor’s analytical properties (linearity, sensitivity, detection limit, reproducibility, etc.) for CGA determination. A linear correlation was observed when the corresponding Δ*I*_2_ values were plotted against varying concentrations ranging from 2.5 × 10^–13^ M to 1.75 × 10^–12^ M (Figure 4). The calibration curve and the corresponding linear regression equation were obtained using least-squares linear regression. This method minimizes the sum of squared residuals between the experimental data points and the fitted line. The high correlation coefficient (*r* = 0.999) indicates an excellent linear fit between the concentration (*C*) and the measured current response (Δ*I*_2_). At 95% confidence intervals, ±0.33 × 10^12^ and ±2.84 were obtained for both slope and intercept, respectively. The residual standard deviation (*s*_*y*/*x*_) was found to be 1.07. The Δ*I*_2_ values were calculated by subtracting the peak current values after template removal and subsequent rebinding at each concentration. The resulting regression equation was determined as follows:

1 $${\Delta I}_{2}\;\left(\mathrm{\mu A}\right)=6.49\times 10^{13}\pm 0.33\times 10^{13}\;\left(\mathrm{\mu A}/M\right)\times C\;\left(M\right)+46.47\pm 2.84\;\left(r=0.999\right)$$

**Figure 4:**
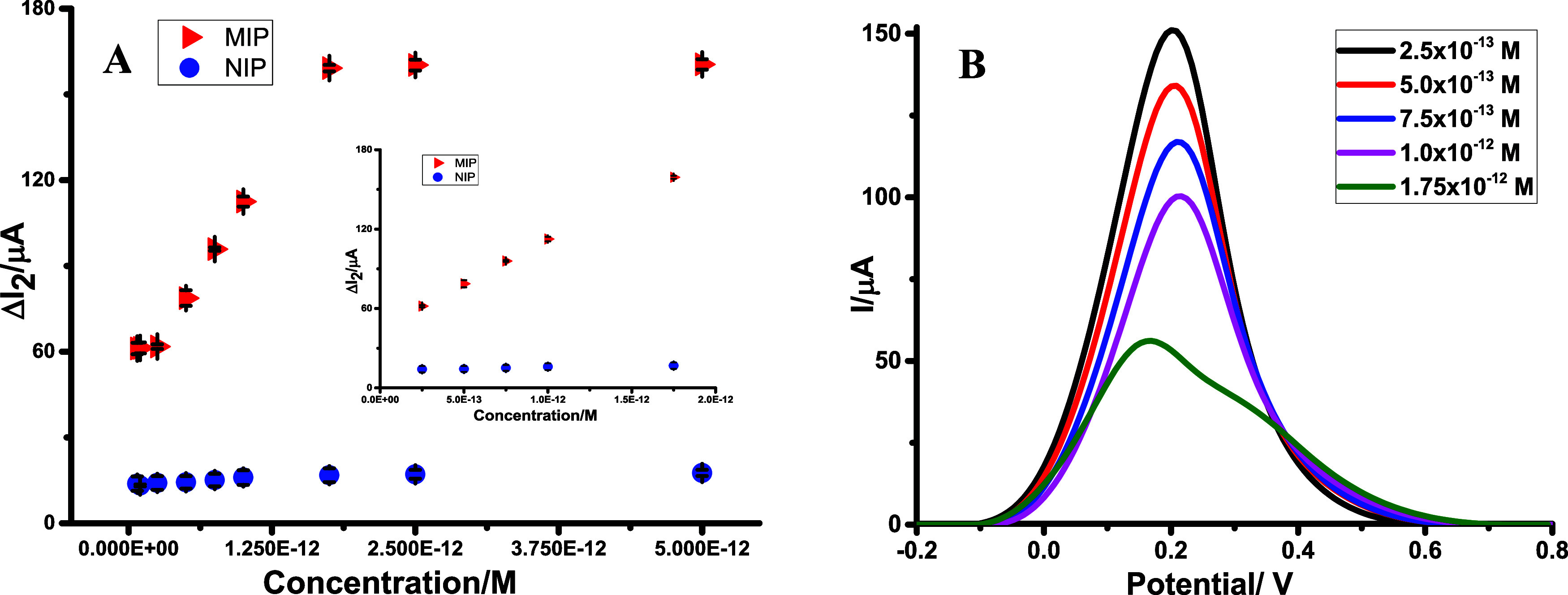
DP voltammograms obtained after rebinding an increasing CGA concentrations standard solution (A); Calibration plots obtained from measurements of increasing CGA concentration using the design CGA/2-TBA@MIP-GCE sensor in standard solutions (B).

Furthermore, the limit of quantification (LOQ) and limit of detection (LOD) were computed in accordance with the formulas provided by the ICH guidelines^24,25^

LOD=3×standarddeviation/slopeandLOQ=10×standarddeviation/slope

The NIP-based sensor did not exhibit a linear electrochemical response within the same concentration range as MIP, as it lacked specific recognition sites. This observation confirms the high selectivity of the developed sensor (CGA/2-TBA@MIP-GCE) toward the target analyte CGA (Figure 4A). The regression equations, correlation coefficients, and other analytical validation parameters are summarized in Table 2. The same sensor configuration was used to evaluate repeatability and reproducibility. Notably, the proposed method detected at lower concentrations, with a lower limit of detection (LOD) and higher sensitivity than previously reported studies (Table 2). As shown in Figure 4B, a significant and systematic decrease in the DPV peak currents of the redox probe was observed as CGA concentrations increased. This decrease is attributed to the increased specific binding interactions with the electrode surface cavities as the CGA concentration rises. The observed current suppression confirms the formation of the analyte-receptor complex and reflects the sensor’s effective recognition and selectivity toward CGA. These results further demonstrate the applicability of the proposed method for the sensitive and reliable quantification of CGA in complex matrices. To evaluate the repeatability and reproducibility of the sensor, ΔI_2_ values were determined by comparing rebinding currents once the effects of repeatability- and reproducibility-related variations were removed, using three different concentrations (5.00 × 10^–13^, 7.50 × 10^–13^, and 1.0 × 10^–12^). In addition, the sensor-to-sensor reusability values were calculated based on the Δ*I*_2_ responses obtained from three independently prepared electrodes at three different concentrations (5.00 × 10^–13^, 7.50 × 10^–13^, and 1.00 × 10^–12^).

**Table 2:** Regression Data of the Calibration Line for CGA@2-TBA/MIP-GCE

Parameter	Standard solution
linearity range/M	2.5 × 10^–13^–1.75 × 10^–12^
slope/μA/M	6.49 × 10^13^
SE of slope	1.03 × 10^12^
intercept/μA	46.47
SE of intercept	0.892
correlation coefficient (*r*)	0.999
LOD/M	2.34 × 10^–14^
LOQ/M	7.79 × 10^–14^
repeatability of peak current (RSD%)^a^	0.53
reproducibility of peak current (RSD%)^a^	1.94
sensor-to-sensor reusability of peak current (RSD%)^a^	2.07

a Each value is the mean of three experiments. The repeatability, reproducibility, and reusability of peak current were calculated for 1.00 × 10^–12^ M.

Relative Standard Deviation (RSD%) values were calculated using the following formula^24,25^

RSD%=100×standarddeviation/average

### 3.5 Analytical Performance of the CGA/2-TBA@MIP-GCE Sensor in Real Samples

The CGA@2-TBA/MIP-GCE sensor was effectively created and assessed for the precise detection of CGA in extracts from *A. limonifolium subsp. Limonifolium, A. limonifolium subsp. pestalozzae, A. linifolium subsp. linifolium, A. linifolium subsp. nallihanicum, and* herbal supplements. The sensor showed outstanding sensitivity, selectivity, and accuracy in detecting CGA in complex matrices (Table 3). The recovery investigations involved adding known amounts of the analyte to samples extracted using various techniques, followed by measurement of the analyte concentration using the CGA@2-TBA/MIP-GCE sensor. The recovery percentages and RSD values from the recovery studies were both within acceptable bounds, making the data highly satisfactory. Table 3 summarizes the results for plant extract and herbal product samples. The initial measurements were conducted without CGA spiking to determine the amount of CGA present in *A. limonifolium subsp. Limonifolium, A. limonifolium subsp. pestalozzae, A. linifolium subsp. linifolium, A. linifolium subsp. nallihanicum, and* herbal supplements made in accordance with the procedure described in Section 2.4. Accordingly, the CGA amounts in each real sample were found to be as follows: Accordingly, the CGA amounts in each real sample were found to be as follows: 2.72, 3.14, 1.377, 33.96, and 30.1 μg g^–1^ for *A. limonifolium subsp. Limonifolium, A. limonifolium subsp. pestalozzae, A. linifolium subsp. linifolium, A. linifolium subsp. nallihanicum*, and herbal supplements, respectively.

**Table 3:** Results of Recovery Experiments for Plant Extracts and Herbal Products

sample	spiked amount/μg. g^‑1^	found amount/μg. g^‑1a^	recovery (%)^a^	RSD%	Bias%
*A. limonifolium* subsp. *limonifolium*	0	2.72	-	1.98	-
	2.50	5.12	98.03	2.98	–1.97
*A. limonifolium* subsp. *pestalozzae*	0	3.14	-	1.24	-
	2.50	5.78	102.45	1.89	3.19
*A. linifolium* subsp. *linifolium*	0	1.37	-	2.49	-
	2.50	3.97	102.82	1.29	2.82
*A. linifolium* subsp. *nallihanicum*	0	3.96	-	1.25	-
	2.50	6.60	102.21	1.41	2.21
herbal supplement	0	30.1	-	1.72	-
	2.50	32.89	100.92	2.07	–0.92

a Each value is the mean of three experiments.

### 3.6 Selectivity Studies

The imprinting factor (*k*) is defined as the ratio of the analytical response obtained from the MIP sensor to that of the corresponding NIP under identical experimental conditions. This parameter is widely used as a quantitative measure of imprinting efficiency and the selectivity imparted by the template molecule. The theoretical concept underlying the imprinting factor assumes that the NIP’s response primarily arises from nonspecific interactions between the polymer matrix and the analyte. In contrast, the response of the MIP results from the combined effect of these nonspecific interactions and the additional specific binding provided by the imprinted recognition cavities. Accordingly, a *k* value greater than one indicates the successful formation of selective and effective binding sites during the imprinting process. The relative imprinting factor (*k*′) is introduced to further normalize the imprinting effect, particularly when comparing sensor responses for different analytes or under varying experimental conditions. This parameter reflects the relative contribution of specific molecular recognition by comparing imprinting factors. In this way, it helps reduce the influence of systematic variations unrelated to the imprinting process, such as differences in electrode surface area or inconsistencies in polymer film coverage.^26–30^ The selectivity performance of the MIP-based CGA/2-TBA@MIP-GCE electrochemical sensor was assessed using imprinting factor (*k*) studies to confirm its molecular recognition of CGA. For this purpose, several structurally similar compounds containing QUE, LUT, GAL, HYP, RUT, p-CA, CAF, API, and NAR were examined as potential competing analytes. The k and relative imprinting factor (*k*′) were calculated using the equations below

2 $$k_{\left(\mathrm{MIP}\right)}=\frac{\Delta I_{2}\;\left(\mathrm{MIP}\right)\;\mathrm{for}\;\mathrm{CGA}}{\Delta I_{2}\;\left(\mathrm{MIP}\right)\;\mathrm{for}\;\mathrm{other}\;\mathrm{drug}}$$

3 $$k_{\left(\mathrm{NIP}\right)}=\frac{\Delta I_{2}\;\left(\mathrm{NIP}\right)\;\mathrm{for}\;\mathrm{CGA}}{\Delta I_{2}\;\left(\mathrm{NIP}\right)\;\mathrm{for}\;\mathrm{other}\;\mathrm{drug}}$$

4 $$k^{\prime }=\frac{k_{(\mathrm{MIP})}}{k_{(\mathrm{NIP})}}$$

The results presented in Table 4 show that the CGA/2-TBA@MIP-GCE sensor exhibits approximately 5–6 times greater affinity and selectivity for CGA than for other molecules.

**Table 4:** Specificity Results of CGA/2-TBA@MIP-GCE with a 1000-Fold Higher Concentration of Similarly Structured Molecules

	MIP/GCE		NIP/GCE		
	Δ*I*/μA	*k*_(MIP)_	Δ*I*/μA	*k*_*(*NIP)_	*k*′_(MIP/NIP)_
CGA	97.01	-	15.1	-	**-**
QUE	17.5	5.54	14.52	1.04	**5.33**
LUT	16.3	5.95	14.7	1.03	**5.79**
GAL	16.8	5.77	14.4	1.05	**5.51**
HYP	16.6	5.84	14.6	1.03	**5.65**
RUT	16.1	6.03	16.1	1.01	**5.95**
p-CA	17.3	5.61	14.46	1.04	**5.37**
CAF	15.8	6.14	14.9	1.01	**6.06**
API	16.7	5.81	15.00	1.01	**5.77**
NAR	16.7	5.81	14.72	1.03	**5.66**

The selectivity of the designed CGA/2-TBA@MIP-GCE sensor was evaluated by measuring its analytical response to 5.0 × 10^–13^ M CGA in the presence of various potentially interfering compounds, which were used to calculate the imprinting factor. As illustrated in Figure 5, the variation in Δ*I*_2_ remained below 4% even when these compounds were present at 1-, 10-, 100-, and 1000-fold excess relative to CGA. Furthermore, the recovery of CGA in these mixtures was consistently high, ranging from 99.4% to 103.9%. These findings indicate that the polymer matrix successfully captures the specific molecular features of CGA, allowing the sensor to selectively discriminate the target analyte from structurally similar or coexisting species. Additionally, the results confirm that the presence of potentially interfering compounds has a negligible effect on the sensor’s analytical performance, demonstrating the robustness and specificity of the molecular imprinting approach for CGA detection. Overall, the data indicate that the CGA/2-TBA@MIP-GCE sensor offers high selectivity and excellent tolerance to interference, critical for reliable measurements in complex sample matrices.

**Figure 5:**
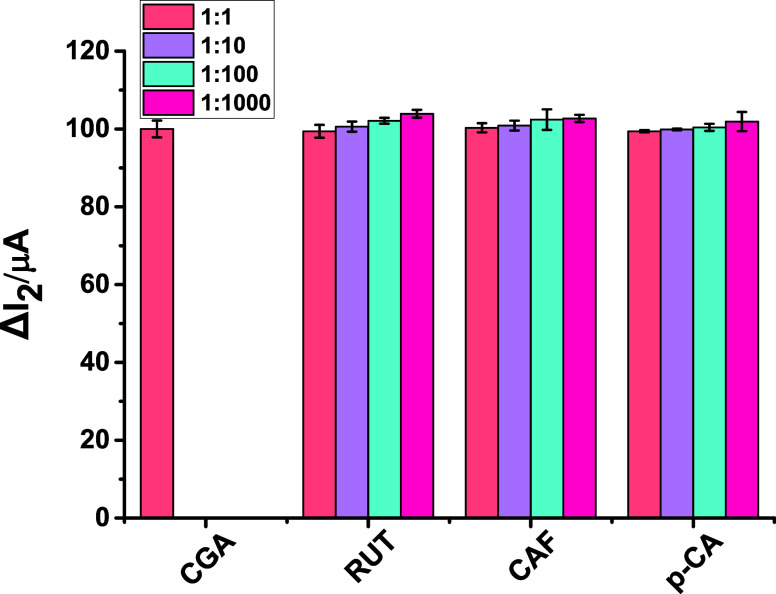
These graphs illustrate the CGA/2-TBA@MIP/GCE sensor in the presence of interfering substances at various ratios: 1:1, 1:10, 1:100, and 1:1000.

### 3.7 Sensor’s Stability and Reusability Tests

To evaluate sensor stability, the modified electrode was stored in a desiccator at room temperature for 1, 3, 5, 7, 10, and 15 days. It was calculated by taking into account the sensor’s performance values obtained on different days. The performance of the sensor developed using the PP method was found to be 98.15% after 3 days, 90.9% after 5 days, 85.9% after 7 days, 76.4% after 10 days, and 73.9% after 15 days. According to these data, the performance of the developed sensor decreases after day 5. As a result, the stability of the CGA/2-TBA@MIP/GCE sensor was found to be 5 days. Furthermore, the reusability of the MIP sensor was also investigated by performing 10 consecutives of rebinding and template removal to evaluate its operational stability during repeated use. As illustrated in Figure 6, the sensor retained approximately 96.12% of its original response after the tenth cycle, demonstrating its strong reliability and consistent performance across multiple uses.

**Figure 6:**
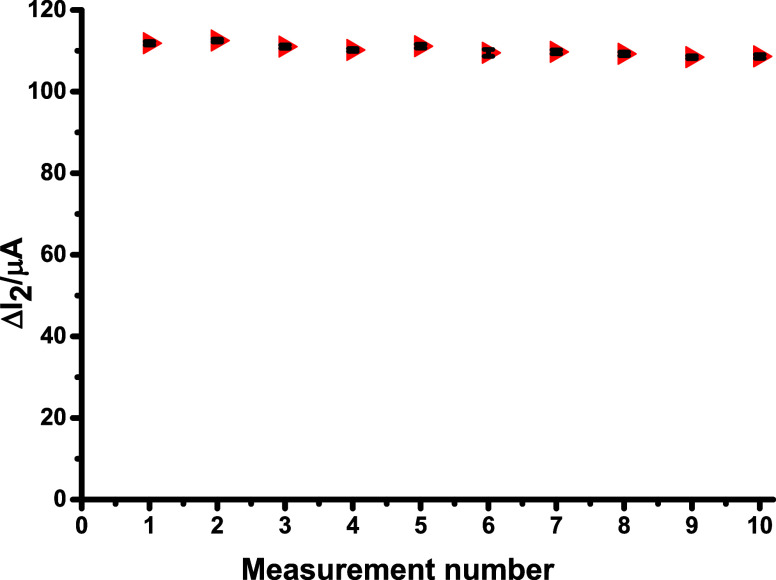
Reusability results of the CGA/2-TBA@MIP/GCE sensor. The rebinding concentration used for the reusability test was 1.00 × 10^–12^ M.

### 3.8 Application to Plant Samples

The developed electrochemical method was applied to determine CGA levels in plant extracts and a commercial drug formulation, and the results were validated against those obtained using the LC–MS/MS reference method. Quantitative results obtained by both approaches are presented in Table 5. The electrochemical method successfully detected CGA in all tested matrices. Statistical comparison using a paired *t* test (p = 0.242) confirmed that there was no significant difference (*p* > 0.05) between the electrochemical and LC–MS/MS results. This demonstrates that the electrochemical platform can produce analytical results comparable to those of the reference LC–MS/MS method.

**Table 5:** Comparison of CGA Quantification by MIP and LC–MS/MS Methods

sample	MIP/M^a^	LC–MS/MS/M^a^
*A. limonifolium* subsp. *limonifolium*	7.68 × 10^–7^	7.77 (±0.35) × 10^–7^
*A. limonifolium* subsp. *pestalozzae*	8.87 × 10^–7^	8.90 (±0.41) × 10^–7^
*A. linifolium* subsp. *linifolium*	3.86 × 10^–7^	3.53 (±0.59) × 10^–7^
*A. linifolium* subsp. *nallihanicum*	1.12 × 10^–6^	1.30 (±0.21) × 10^–6^
herbal supplement	8.47 × 10^–6^	8.88 (±0.10) × 10^–6^

a Mean ± standard deviation

### 3.9 Performance Comparison of Selected Analytical Techniques

Table 6 presents a thorough summary of the various analytical techniques used in relation to the analytical performance of CGA. A detailed review of the existing literature highlights several drawbacks associated with spectroscopic and chromatographic methods. These include the widespread use of organic compounds, the need for multistage sample pretreatment before measurement, the relatively long analysis time, and the chemicals used, which are hazardous or toxic and pose environmental and health risks. The CGA/2-TBA@MIP-GCE sensor demonstrated superior selectivity and sensitivity compared with conventional methods. The experimental results indicate that the proposed sensor offers more than effective performance, providing notable advantages in usability, cost-efficiency, practical applicability, and environmental sustainability. In addition, the new method’s demonstrated excellent linearity, repeatability, low detection limits, selectivity, and stability make it a viable choice for sensitive and reliable analysis across various disciplines.

**Table 6:** Comparison of Previous Studies on CGA Determination with This Study

method	linear range	LOD	sample	recovery	refs
reverse-phase rapid resolution liquid chromatography (RP-RRLC) with diode-array detection (DAD)	12.33–143.50 μg mL^–1^	0.29 pg mL^–1^	green coffee	97.87–106.67%	^31^
liquid chromatography–ultraviolet detection (LC-UV)	0.001–13.5 μg mL^–1^	1.0 ng mL^–1^	rat plasma	90.8–119.8%	^32^
reversed-phase high-performance liquid chromatography with diode array detection (RP-HPLC-DAD)	16.67–83.33 μg mL^–1^	1.64 μg mL^–1^	*Lonicera japonica* flowering buds	103.98–108.63%	^33^
electrochemical sensor based on a nanostructured platform and molecularly imprinted sol–gel film	0.08–500 μmol L^–1^	0.032 μmol L^–1^	coffee, tomato, and apple	99.3–108.6%	^34^
ultrasensitive fluorescence	0.01 to 0.1 μM and 0.1 to 20.0 μM	8.87 nM and 0.12 μM	honeysuckle, green coffee beans, roasted coffee beans, instant coffee	honeysuckle: 98.9–106.7%, green coffee beans: 99.5–102.5%, roasted coffee beans: 99.7–100.4%, instant coffee: 99.6–100.8%	^35^
high-performance thin-layer chromatography (HPTLC) and high-performance liquid chromatography (HPLC)	HPTLC: 0.05–0.25 mg mL^–1^	HPTLC: 3.5 μg L^–1^	green coffee bean extracts (six commercial samples)	HPTLC: 97.08–99.02%	^36^
	HPLC: 85–250 μg mL^–1^	HPLC: 10 μg mL^–1^		HPLC: 98.80–103.10%	
core–shell-structured electrochemical sensor (Fe3O4@MIL-100(Fe))	0.1–460 μmol L^–1^	0.05 μmol L^–1^	apple extract, coffee drink, cold medicine tablets	96.0–103.8%	^37^
HPLC-DAD	10–1000 mg L^–1^	-	coffee samples	-	^38^
HPLC-WVD	5–200 mg L^–1^	0.1–1 mg L^–1^	green coffee bean samples	-	^39^
RP-HPLC	0.005–0.05 mg mL^–1^	0.0028 mg mL^–1^	*Morus alba* leaves	94.84%	^40^
**CGA/2-TBA@MIP-GCE**	**2.5 × 10** ^**‑13**^–**1.75 × 10** ^**‑12**^ **M**	**2.34 × 10** ^**–14**^ M (23.40 fM)	***A. limonifolium* subsp. *limonifolium*, *A. limonifolium* subsp. *pestalozzae*, *A. linifolium* subsp. *linifolium*, *A. linifolium* subsp. *nallihanicum*, *and* herbal supplements**	**98.03%**	**this Study**
				**102.45%**	
				**102.82%**	
				**102.21%**	
				**102.29%**	

## 4 Conclusion

CGA, a potent antioxidant found in plants, vegetables, and fruits, is one of the most common phenolic compounds and has demonstrated numerous pharmacological activities. The challenge for researchers is to develop selective and sensitive sensors for the various applications of CGA-containing extracts, given their complex structures and importance. This work reports the development of a novel electrochemical sensor based on molecular imprinting for the sensitive and selective detection of CGA in plant extracts and herbal supplements. The sensor design used 2-TBA as the functional monomer, and the polymer layer was constructed on the GCE surface via the PP method. The developed CGA/2-TBA@MIP/GCE sensor was characterized electrochemically and morphologically using CV, EIS, and SEM to elucidate its surface morphology and interfacial properties. The linear range of the sensor was found to be 2.5 × 10^–13^ – 1.75 × 10^–12^ M, and the LOD was calculated to be 2.34 × 10^–14^ M. In conclusion, the proposed CGA/2-TBA@MIP/GCE sensor exhibits outstanding selectivity and sensitivity toward CGA, along with satisfactory reproducibility, stability, and fast response behavior, making it a reliable and efficient analytical tool for the determination of CGA in complex plant-derived and commercial herbal supplement matrices.
